# Protocol: systematic review of the association between socio-economic status and survival in adult head and neck cancer

**DOI:** 10.1186/s13643-017-0545-0

**Published:** 2017-08-02

**Authors:** Bilal G. Taib, Joseph Rylands, Sue Povall, Terry M. Jones, David Taylor-Robinson

**Affiliations:** 1 Postgraduate Centre, Royal Liverpool University Hospital, Prescot Street, Liverpool, L7 8XP UK; 2 Aintree University Hospital, Longmoor Lane, Liverpool, L9 7AL UK; 3 Institute of Psychology, Health and Society, University of Liverpool, Waterhouse building, Liverpool, L69 3BX UK; 4 Department of Molecular and Clinical Cancer Medicine, University of Liverpool, 200 London Road, Liverpool, L3 9GA UK

**Keywords:** Head and neck cancer, Socio-economic status, Socio-economic inequalities, Survival, Systematic review, Meta-analysis

## Abstract

**Background:**

Head and neck cancer incidence is increasing worldwide. Despite overall improvements in survival, numerous studies suggest worse survival in more disadvantaged populations; however, this literature has not been systematically reviewed. The aim of this review is to investigate whether lower compared to higher socioeconomic status (SES) influences survival in head and neck squamous cell cancer (HNSCC) and explore possible explanations for any relationship found.

**Method:**

A systematic strategy will be used to identify articles, appraise their quality and extract data. Online databases including MEDLINE, Web of Knowledge, ESBCO Host and Scopus will be used to locate observational studies of adults with a primary diagnosis of head and neck cancer in EU15+ countries (15 members of the EU, Australia, Canada, Norway, USA and New Zealand) where the outcomes report associations between SES and survival. This will be augmented by searching for grey literature and through reference lists. Data will be extracted using a standardised form. Study quality will be assessed using the Newcastle Ottawa scale and where possible meta-analysis of the pooled data will be conducted.

**Discussion:**

This review will quantify the association between SES and survival outcomes for adult head and neck cancer patients in developed countries. The results will help identify gaps in the literature and therefore direct further novel research in the field. Ultimately, this will inform public policy and strategies to reduce the inequalities in HNSCC survival.

**Systematic review registration:**

PROSPERO CRD42016037019.

**Electronic supplementary material:**

The online version of this article (doi:10.1186/s13643-017-0545-0) contains supplementary material, which is available to authorized users.

## Background

A large body of literature on health inequalities shows that more disadvantaged populations are more likely to experience illnesses and premature death [1]. Socioeconomic inequalities have been demonstrated in head and neck cancer incidence (HANC) [2]. However, evidence on the relationship between social deprivation and HANC survival has not been comprehensively synthesised.

Collectively, head and neck cancer is the 6th most common cancer worldwide accounting for 550,000 new cases per annum and this figure is rising [3, 4]. Overall and disease-specific survival for head and neck cancer patients has improved, particularly for human papillomavirus (HPV)-positive oropharyngeal cancer [5, 6]. However, the full extent of this survival benefit has not been conferred to HPV-negative head and neck squamous cell carcinomas (HNSCC) which tend to affect a different cohort of patients [7, 8].

In 2013, over 8000 new cases of HNSCC were diagnosed in England and Wales with an overall mortality rate of 13.4% [9]. The estimated cost of HANC hospitalisations in the NHS is around £57.1 million per annum [10]. This debilitating disease also impacts on daily life long after the initial diagnosis and treatment. In the USA, the loss of productivity, as measured by a loss of earnings, is estimated to be $3.4 billion [11]. The economic burden and social burden of HANC falls hardest on those in lower socio-economic status (SES) groups.

There are several individual studies examining the link between SES and survival in head and neck cancer in general and or at specific subsites. Most suggest there is a strong association between low SES and worse survival outcomes [12–21]. Using Scottish registry data, Robertson et al. estimated that there was a 27% greater risk of death for those in the most deprived SES groups compared to the least [12]. Nutting et al. described that improvements in laryngeal cancer survival in England and Wales for men from 1985 to 2001 are limited to the most affluent groups [22]. Furthermore, both McDonald et al. and Rachet et al. have suggested that the absolute differences in survival between the most and least affluent groups appear to be increasing [13, 14].

Estimates of the impact of SES on health and neck cancer survival vary. This may be because studies have used different designs, measured SES in different ways and adjusted for different confounding variables (such as age, sex, race, stage, alcohol and tobacco consumption, region, income, education and occupation). For instance, a retrospective cohort study collating evidence from two oncology studies on non-metastatic head and neck cancer by Coyne et al. estimates that the lowest income groups have a 30% increased risk of HNSCC mortality whereas a similar study looking into neighbourhood deprivation in America and its effect on head and neck cancer outcomes published by Retizel et al. 2012 estimates this to be at 70% [20, 23]. In this review, we aim to synthesise the available evidence and to explore reasons for any differences uncovered across the included studies.

**Fig. 1:**
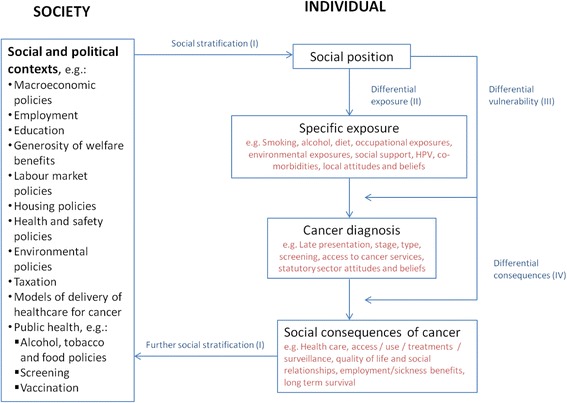
Diderichsen model of pathways to health inequalities applied to HNSCC cancer incidence and outcomes [24]

Our review is part of a larger project aimed at better understanding the relationship between SES and a range of HNSCC outcomes. Our analyses are informed by the Diderichsen model of pathways to inequalities in health (Fig. 1) [24]. In the model, an individual’s social position leads to differential exposure and vulnerability to risk factors, for instance tobacco and alcohol in the case of HNSCC. These processes lead to differential health outcomes with varying levels of access to health care that may have differential social consequences on income and ultimately survival. Similar frameworks have been utilised to describe inequalities in cancer outcomes in breast, prostate, cervical and colorectal cancer [25, 26].

The first part of this pathway, the association between SES and the risk of developing HANC, has been explored in a systematic review by Conway et al. The review showed that people in the lowest educational and income groups had at least a twofold increased risk of developing HANC [2].

However, there has been no overarching systematic review of the evidence assessing the association between SES and survival outcomes in HNSCC, and only parts of the putative causal pathway (as per Fig. 1) have been explored. The aim of this systematic review is therefore to investigate whether lower compared to higher SES influences survival in HNSCC in developed countries and to explore the variables that may explain any relationship found. We will assess the magnitude, statistical significance and associations of the SES variables with survival. This will help identify any gaps in the current evidence base and allow us to focus our public health efforts to reduce the burden of HNSCC.

## Methods/design

To improve the transparency and completeness of this systematic review protocol, a completed copy of the Preferred Reporting Items for Systematic Reviews and Meta-Analyses for Protocols 2015 (PRISMA-P 2015) checklist is available as an Additional file 1 [27].

### Research question

For individuals from developed countries, does lower compared to higher socioeconomic status influence survival in head and neck cancer?

### Population

Any individual including and over the age of 18, of any gender from a European Union 15+ (EU15+) country will be selected. A EU15+ country is any of the first 15 members of the EU and Australia, Canada, Norway, USA and New Zealand. A similar set of countries has been used in previous UK-based comparative health inequality studies [28].

### Outcome

The primary outcome of interest is differences in HNSCC survival between SES groups. This aggregate level data is measured as univariate and multivariate odds ratios or hazard ratios with 95% confidence intervals.

### Exposure

We will focus on SES measures at individual or aggregate level data by income, education, occupation and neighbourhood deprivation indices. SES levels will be measured as per Conway et al. using a five-level ordinal strata, if possible [29].

### Other data

In addition, data on potentially confounding or mediating factors will be sought, such as alcohol and smoking, age, sex, marital status, co-morbidities, time to presentation, stage of presentation and types of surgical and or medical interventions.

### Inclusion/exclusion criteria

The following studies will be included in the review:

- ○ Observational studies (cross-sectional, ecological, case-control, cohort (prospective and retrospective) reporting quantitative results and analysis of empirical data on association between socioeconomic status and survival for patients with HNSCC (ICD C00-C14 and C30-C32).
- ○ This can be measured at individual or aggregate level by occupation, income, education, employment or neighbourhood deprivation where the outcome is an odds ratio or hazards ratio with measures of variance.
- ○ EU15+ countries.
- ○ Publications written in or translated into English, reporting on human subjects and published from 1990, inclusive.

The following studies will be excluded from the review. Those focussing on:

- ○ Loco-regional recurrence of head and neck cancer
- ○ Second primary HNSCC
- ○ Outcomes for patients primarily presenting with cancers of the nasopharynx, thyroid or oesophagus
- ○ Subjects <18 years of age

### Search strategy

A systematic strategy will be used to identify relevant articles, assess their eligibility and appraise their quality. This will be achieved by searching databases, the reference lists for any studies accepted for inclusion and grey literature.

Electronic searching of four databases will be performed: MEDLINE, Web of Knowledge, ESBCO Host and Scopus. These databases were discussed within the research collaboration and were identified as the most likely to yield relevant papers. We will include data from the 20 economically developed EU 15+ nations outlined in Appendix 1.

The search terms outlined in Appendix 2 were piloted prior to selection. They comprise of specific head and neck cancers, inequality terms and developed countries of interest. Relevant terms were identified during an initial scoping literature review of the topic.

Where possible, terms will be exploded to widen the search. Terms will be added as keywords if they cannot be exploded or if the exploded terms are not relevant to the research question. Truncation and proximity operators will also be applied as necessary to broaden the search. For consistency, the same terms will be inputted for each database, e.g. Scopus and Web of Science; however, as the functionality of each database is different, the terms have been adapted for correct use in each.

The same filters will be applied to all included databases as per the inclusion criteria. This will ensure articles are in English, on human subjects and investigating the influence of SES on HNSCC from 1990 onwards. As social conditions change over time, limiting the literature to this time period will ensure that publications are as relevant as possible to the present day. The search will be broad as studies examining the incidence of HNSCC may also examine the impact of SES on survival as a secondary outcome. The searches will be re-run after the initial data extraction period so any further studies are included. The remaining publications will then be exported to a referencing software and combined so that any duplicates are removed. These will then be screened as per the eligibility criteria.

Titles, abstracts and full texts will be screened independently by one author (BGT). The second reviewer will screen a 10% sample of the extracted papers—at title, abstract and full text stages (SP) to ensure consistency in the application of the eligibility criteria. If it is not clear from the title or abstract whether analyses by SES have been performed, the full text of the article will be retrieved and examined. All full text studies will be reviewed independently by two reviewers (BGT and SP) to ensure that the studies meet the inclusion and exclusion criteria. Any discrepancies either at the title, abstract or full-text phase will be discussed (DTR and TMJ) until an agreement is reached between all reviewers.

Another strategy will consist of searching through the reference list of articles that may have been missed by electronic database searches. Studies of interest will have their titles and abstracts analysed and screened as per our inclusion and exclusion criteria. We will also use the forward citation map to locate newer articles which have cited the older study identified. The full text for those articles that meet the initial inclusion criteria will be screened (BGT and JR) and any discrepancies discussed between the reviewers.

Finally, we will further augment our search by searching for grey literature. We will do this by entering the terms “head and neck cancer”, “socioeconomic”, “social class” and “deprivation” into the Google internet search engine and Google Scholar search application and assessing the first 100 results including reports from cancer registries, HANC audit reports, published abstracts and theses. In a similar manner, we will also search OpenGrey a repository for grey literature in Europe. Again, this will be performed independently by two reviewers (BGT and JR), and any disagreements will be resolved through discussion with the wider group.

### Quality assessment

Risk of bias and quality assessment will be conducted by two reviewers (BGT and JR). Methodological quality of the included studies will be assessed using the Newcastle-Ottawa Scale and/or Cochrane risk of bias framework [30, 31]. Any discrepancies identified will be discussed and reviewed by the review team.

### Data analysis and synthesis

To organise this data and to aid comparison between studies, tables will be created from which extracted data will be placed in a standardised manner. A proforma has been developed and piloted on three studies. Information to be extracted includes: year of publication, country, hypothesis, study design, level of analysis, sample size, age, recruitment period, SES variable measured, health measure outcome(s), tool used to measure the outcome, covariates analyses, significant and non-significant results, adjusted and unadjusted odds ratio/hazard ratio (OR/HR) for all cause and HNSCC-specific mortality, confidence intervals, conclusions, comments on limitations and quality assessment. Extracted data will be checked by one other reviewer (JR). We are anticipating to be able to explore heterogeneity and undertake analyses to explore the impact of potentially moderating factors such as age, sex, performance status, site, alcohol consumption, smoking, grade, stage, treatment employed, SES measures and neighbourhood deprivation. Harvest plots maybe used where appropriate to display and summarise the results of the studies and to display the impact of moderating factors subgrouping graphically [32].

Where possible meta-analysis of extracted data will be undertaken via the Mantel-Haenszel method using a fixed-effects model as default, unless significant heterogeneity is present, in which case a random-effects model will be used. HRs will be calculated with 95% confidence intervals. A *p* value of <0.05 will be regarded as significant. Heterogeneity will be assessed using the *I*-squared statistic. We will undertake sensitivity analysis on the basis of study quality.

### Dissemination

The systematic review will be submitted for publication. The findings will be presented at national and international conferences. It will also be presented locally to help inform the council and public health initiatives.

## Discussion

This protocol describes how this systematic review will determine the impact of SES on the survival outcomes of adult HNSCC patients. Using the Diderichsen model (Fig. 1), we will identify the key variables and pathways that generate inequalities in survival in HANC.

In the UK, the gender-specific inequality gaps in survival between certain HANCs are the widest of any cancer [14]. Furthermore, the incidence of HANC is increasing and stark geographical inequalities exist in the UK [29]. This, coupled with a recent survival benefit generally limited to the more affluent groups, highlights the necessity of better understanding of SES and HNSCC [22].

We hope that the results of this review will identify potential targets for intervention to reduce inequalities in HNSCC outcomes.

## Appendix 1

**Table 1:** List of the EU 15+ countries included in the study

European	Other countries
Austria	Australia
Belgium	Canada
Denmark	New Zealand
Finland	Norway
France	USA
Germany	
Greece	
Ireland	
Italy	
Luxembourg	
Netherlands	
Portugal	
Spain	
Sweden	
UK	

## Appendix 2

**Table 2:** MEDLINE search strategy

Inequality	Head and neck cancers	Survival
“Socioeconomic Factors” [Mesh]	“Head and Neck Neoplasms” [Mesh]	survival
inequit*	“Carcinoma, squamous cell of head and neck” [Supplementary Concept]	
inequalit*	head AND neck AND (cancer OR neoplasm OR carcinoma)	
disparit*		

(“Socioeconomic Factors” [Mesh] OR inequit* OR inequalit* OR disparit*) AND (“Head and Neck Neoplasms”[Mesh] OR “Carcinoma, squamous cell of head and neck” [Supplementary Concept] OR (head AND neck AND (cancer OR neoplasm OR carcinoma)) AND survival); limited to >1989, human, abstract available, English language. Rows combined with logical “OR” and columns with logical “AND”

## Additional file

Additional file 1: PRISMA-P checklist. (DOC 81 kb)

## Abbreviations

HANC: Head and neck cancer
HNSCC: Head and neck squamous cell carcinomas
SES: Socio-economic status
